# Region of interest determination algorithm of lensless calcium imaging datasets

**DOI:** 10.1371/journal.pone.0308573

**Published:** 2024-09-17

**Authors:** Virgil Christian Garcia Castillo, Latiful Akbar, Ronnakorn Siwadamrongpong, Yasumi Ohta, Mamiko Kawahara, Yoshinori Sunaga, Hironari Takehara, Hiroyuki Tashiro, Kiyotaka Sasagawa, Jun Ohta

**Affiliations:** 1 Graduate School of Science and Technology, Nara Institute of Science and Technology, Ikoma, Japan; 2 Department of Health Sciences, Faculty of Medical Sciences, Kyushu University, Fukuoka, Japan; Università di Pisa: Universita degli Studi di Pisa, ITALY

## Abstract

Advances in fluorescence imaging technology have been crucial to the progress of neuroscience. Whether it was specific expression of indicator proteins, detection of neurotransmitters, or miniaturization of fluorescence microscopes, fluorescence imaging has improved upon electrophysiology, the gold standard for monitoring brain activity, and enabled novel methods to sense activity in the brain. Hence, we developed a lightweight and compact implantable CMOS-based lensless Ca^2+^ imaging device for freely moving transgenic G-CaMP mouse experiments. However, without a lens system, determination of regions of interest (ROI) has proven challenging. Localization of fluorescence activity and separation of signal from noise are difficult. In this study, we report an ROI selection method using a series of adaptive binarizations with a gaussian method and morphological image processing. The parameters for each operation such as the kernel size, sigma and footprint size were optimized. We then validated the utility of the algorithm with simulated data and freely moving nociception experiments using the lensless devices. The device was implanted in the dorsal raphe nucleus to observe pain-related brain activity following a formalin test to stimulate pain. We observed significant increases in fluorescence activity after formalin injection compared to the control group when using the ROI determination algorithm.

## 1. Introduction

Calcium imaging (Ca^2+^ imaging) is extensively utilized in neuroscience to study the brain. Using genetically encoded calcium indicators (GECI), changes in calcium concentration caused by action potentials can be detected and linked to spontaneous animal behavior or experimental stimulation. Fluorescence microscopes are typically used in Ca^2+^ imaging to observe neural activity at high spatial resolutions [1]. However, the bulkiness of these microscopes forces the mouse to be fixed with the equipment and not able to behave freely. Additionally, light scattering properties of the brain restricts imaging depth to only ~1 mm [2].

To observe naturalistic mouse behavior, Schnitzer’s group developed the first head-mountable miniaturized fluorescence microscope [3]. Using their device, concurrent Ca^2+^ spikes of over 200 neurons from 9 different cerebellar microzones were recorded and synchronization between individual microzones was observed. Since then, multiple groups have created their versions of what came to be known as the miniscope. Additionally, with the help of Gradient INdex (GRIN) lenses, even deep brain regions such as the central amygdala (4.6 mm) [4] can be imaged by the miniscope [5, 6]. Recent advances include decreasing device form factor (smallest: 11×11×18 mm, 1.6 g), incorporating multiphoton microscopy, integrating optogenetic capability, implementing wireless connectivity and mounting two devices for dual region recordings [5–12]. However, these devices typically weigh more than 2 g which is 8% of the usual weight of an adult mouse. Additionally, GRIN lens implantation entails considerable tissue damage due to its size.

Another widely used *in vivo* fluorescence imaging technique is fiber photometry which measures Ca^2+^ activity with time-correlated single-photon counting (TCSPC)-based fiber optics [13]. Optical fibers are typically small and flexible which minimizes implantation tissue damage but comes at the cost of being unable to measure single cell activity. Resolution enhancements have been reported with multimode optical fibers with a holographic modulator [14] or two-photon endoscopy [15]. Neurotransmitter detection has also been reported with gold nanoisland-decorated tapered optical fibers for surface enhanced Raman spectroscopy [16]. However, optical fibers can impose movement restrictions on mice due to their rigidity as compared to an electrical wire [1].

In this regard, our laboratory previously reported on an ultralight and compact CMOS-based microimaging device that weighs only 0.02 g and measures 200 μm in thickness. The device is considerably lighter than miniscopes and is connected by electrical wires enabling more naturalistic mouse movements. In addition, in contrast to the axial plane imaging of the miniscope and fiber photometry, the CMOS device enables simultaneous imaging of multiple brain layers with a single device because imaging is in the sagittal plane. Owing to its size and weight, multi-region observations have been possible through dual-device implantation [17] or integration with 1 or 2 microdialysis probes [18, 19]. However, this miniaturization was achieved at the cost of the device’s lens system. The Bio-Flatscope is another lensless Ca^2+^ imaging system that utilizes phase-masks to enable image reconstruction [20]. However, applications have been limited to head-restrained mice and brain surface imaging. Without image reconstruction in a lensless system, images are not clearly resolved [21] so identification of regions of interests (ROIs) has been difficult. ROIs are useful for localization of different fluorescence activity patterns which can be further mapped to the brain. Additionally, ROI selection can segregate pixels into active and non-active (no neural activity) pixels which decreases noise in Ca^2+^ traces. Hitherto, ROI selection has been based on visual inspection of the brightest regions [17, 18, 22], but this method can cause low intensity activity to be missed. Besides, ROI selection can be very inconsistent due to personal bias. For this reason, an automatic ROI determination method needs to be developed to improve localization of fluorescence activity and enhance signal quality.

The most widely used Ca^2+^ imaging ROI selection method is constrained non-negative matrix factorization (CNMF) which decomposes imaging datasets into a two-dimensional matrix representing the cells’ spatial position and one-dimensional matrix representing their fluorescence activity [23]. CNMF improved over the non-negative matrix factorization of Maruya *et al*. [24], and independent component analysis of Mukamel *et al*. [25], by imposing constraints on temporal dynamics, cell sparsity and cell compactness. Another widely used techniques is Suite2p [26] which uses a dictionary learning approach to represent and reconstruct Ca^2+^ imaging data. Other methods utilize correlations between pixels [27] or their low-frequency components spectral components [28]. Although ROI selection methods already exist for Ca^2+^ imaging, these methods were optimized for lens-based system and are, consequently, difficult to use on videos taken with lensless devices.

In this work, we developed an ROI selection algorithm for the lensless imaging device based on adaptive binarization. Otherwise known as local thresholding, adaptive binarization sets a different threshold value for each pixel in an image depending on the values of surrounding pixels. Adaptive binarization was applied to a specified number of frames and then denoised using morphological image processing. The average binarized frame was calculated and then the same set of operations were used to arrive at a final binarized image. The effect of the parameters of each operation such as sigma value, kernel size and opening footprint was studied. The effectiveness of the algorithm at marking ROIs was then demonstrated with simulated datasets and recordings of freely moving mouse experiments.

## 2. Methods

### 2.1. Device fabrication

**Fig 1:**
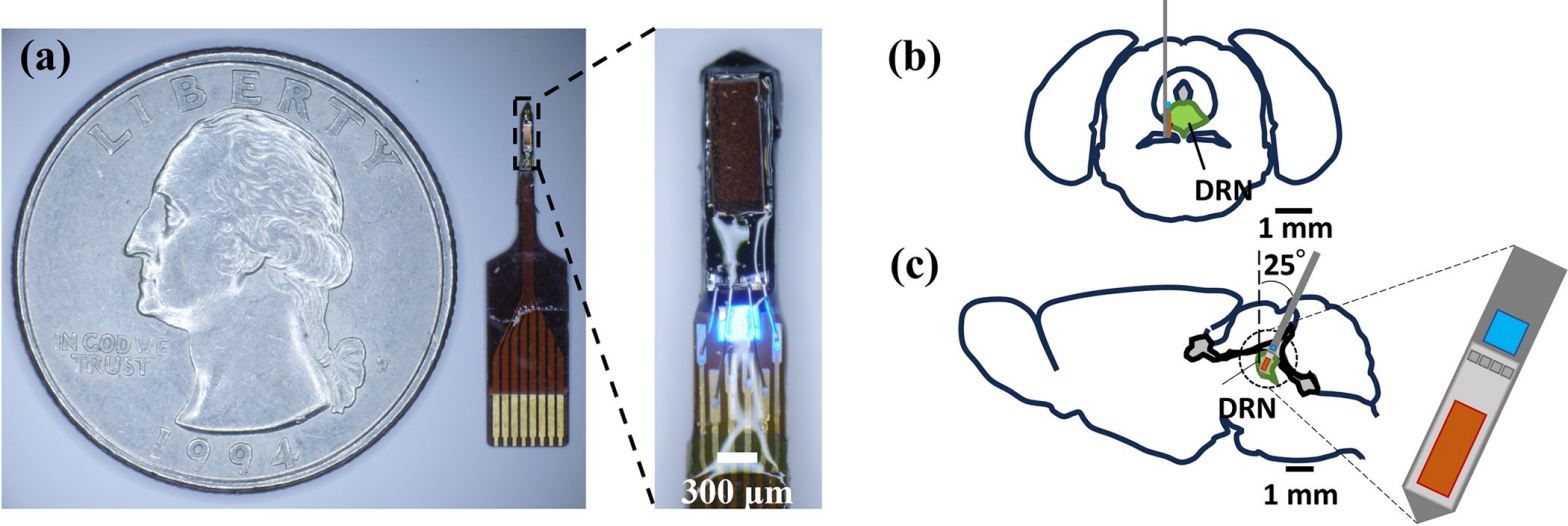
Image of CMOS-based device and implantation schematic. (a) Photograph of implantable CMOS-based imaging device. (b) Coronal (-4.7 mm) and (c) sagittal (0.12 mm) brain slice schematic of device implantation into the DRN.

A CMOS imaging chip with an imaging area of 120 x 40 pixels and a micro-LED with center wavelength 470 nm (ES-VEBCM12A, EPISTAR Corporation, Taiwan) were fixed on a polyimide flexible printed circuit (FPC) substrate (Taiyo Technorex Co., Ltd., Wakayama, Japan) by epoxy resin (Z-1; Nissin resin, Yokohama, Japan). Afterward, electrical contacts of the chip and LED were wire bonded (7400C-79, West Bond, Anaheim, CA, USA) with aluminum wires (TANAKA Holdings Co., Ltd., Tokyo, Japan) to the FPC. Then, an absorption filter was deposited on the imaging area and fixed by heating in a vacuum oven for 2 hours at 120°C. The absorption filter is a dye dissolved in a thin film of photopolymer designed to absorb excitation light and, thus, block contamination from the excitation light. The film was fabricated by embedding a yellow dye (Valifast^®^ yellow 3150 dye, Orient Chemical Industries, Osaka, Japan) in a photopolymer (Norland Optical Adhesive 63, Cranbury, NJ, USA) film. A 1:1:1 by mass solution of the dye, cyclopentanone and photopolymer was mixed vigorously using a vortex mixer then spin coated on a silicone substrate. The film was then cured by heating at 90°C for 30 minutes and exposing to UV for 30 seconds. Finally, the absorption filter was patterned by Nd:YAG laser (Callisto VL-C30RS-GV, TNS Systems) before deposition to match the CMOS imaging area. Leakages in the filter were sealed by black resist resin containing a 1:1:1 ratio of SR-3000L, SG-3000L, and SB-3000L (Fujifilm, Tokyo, Japan) before protecting the aluminum wires with epoxy resin [22]. Finally, the device was coated with approximately 2.5 μm of parylene-C (SCS Labcoter^®^ 2). A photograph of the completed device with a schematic of its implantation in the dorsal raphe nucleus (DRN) of the mouse brain is shown in Fig 1.

### 2.2. Animals

This study was conducted in strict accordance with the Nara Institute of Science and Technology’s Regulations for the Conduct of Animal Experiments. The protocol was approved by the NAIST Animal Experiment Ethics Committee (Animal Experimental Plan Approval Number: No. 2402). All surgeries were performed under triple anesthesia <medetomidine hydrochloride/midazolam/butorphanol tartrate>, followed by recovery from anesthesia with a medetomidine antagonist. After the experiment, the animals were anesthetized with 10% urethane and perfused with paraformaldehyde. Every effort was made to minimize suffering.

Transgenic G-CaMP6 mice, strain FVB-Tg (Thy1-GCaMP6)5Shi (RBRC09452, RIKEN) maintained in the NAIST Animal Facility were used in these experiments. Mice were kept in a 12-hr light/dark cycle and given ad libitum access to food and water. Six transgenic mice were used in this study divided into two groups of 3: formalin-injected and PBS-injected (control).

### 2.3. Surgery and device implantation

Typically, a mouse was anesthetized with a cocktail of medetomidine, midazolam and butorphanol at a mass ratio of 0.3:4:5 [29] and then scalp fur were trimmed before fixing the mouse onto a stereotaxic apparatus with an ear bar. A heating pad was used to help maintain the mouse’s body temperature. Next, the scalp was disinfected with chlorhexidine gluconate and incised along the midline to expose the cranium. A burr hole was then drilled for implanting CMOS imaging devices to the dorsal raphe nuclei (DRN). Next, devices were implanted to the DRN [-5.56 mm anteroposterior (AP), 0.35 mm mediolateral (ML), 3.31 mm dorsoventral (DV) relative to the bregma, tilted 25° anteriorly]. The device was then fixed using dental cement which also served as protection for the incision. Finally, the mouse was injected with atipamezole hydrochloride to reverse the anesthesia and placed in an acrylic cage with access to food and water.

### 2.4. Formalin injection and imaging experiment

Imaging experiments were conducted one day after device implantation. Typically, a mouse was anesthetized with isoflurane to attach the implanted microimaging device to a custom-made imaging system called CIS_NAIST. The mouse was then returned to its cage and given time to recover from the anesthesia. The cage was placed in a dark room where illumination only comes from blue light to prevent interference as blue light is blocked by the device’s absorption filter. The recording was then started at a framerate of approximately 10 fps. After about 1 hour and once the fluorescence signal had stabilized, the mouse was injected with 2% paraformaldehyde in the dorsal part of its right hind paw. The recording continued until over 60 minutes of data was accumulated. For the control group, phosphate buffered saline was injected instead of paraformaldehyde. After each experiment, the mouse was sacrificed by transcardiac perfusion of saline followed by 4% paraformaldehyde.

### 2.5. Data processing

The start and end of the duration procedure was marked in the imaging data. The data was trimmed into approximately 15 minutes before the stimulation and 60 minutes after. These two segments were then concatenated. The frames before stimulation were designated as baseline, F_0_.

Before ROI selection, each pixel was baselined to %ΔF_*i*_/F_*i*,0_ as expressed in Eq 1 where F_*i*,*t*_ is the fluorescence intensity of the *i*^*th*^ pixel at time point *t* and F_*i*,0_ is the average baseline fluorescence of the i^th^ pixel.


$$\%\frac{\Delta F_{i}}{F_{i,0}}=\frac{F_{i,t}-F_{i,0}}{F_{i,0}}\times 100\%$$ Eq 1

After ROI selection, ROI traces were calculated as shown in Eq 2 where ${\bar{F}}_{t}$ is the average fluorescence of all pixels in the ROI and F_0_ is the average baseline fluorescence of all pixels in the ROI.


$$\%\frac{\Delta F}{F_{0}}=\frac{{\bar{F}}_{t}-F_{0}}{F_{0}}\times 100\%$$ Eq 2

All data processing was conducted in Python 3.10.6 (http://www.python.org/, accessed on 10 October 2023) using Spyder IDE 5.4.3 on a desktop computer equipped with an Intel(R) Core(TM) i7-9700 processor running at 3.00GHz, 32 GB of RAM and 512 of SSD storage. Statistical testing and short-time Fourier transform were done with SciPy 1.11.3 [30]. Morphological image processing and adaptive binarization were performed with scikit-image 0.22.0 [31]. Principal components analysis, k-means clustering and silhouette score calculation were implemented with scikit-learn 1.3.1 [32].

## 3. Results

### 3.1. Parameters optimization

**Fig 2:**
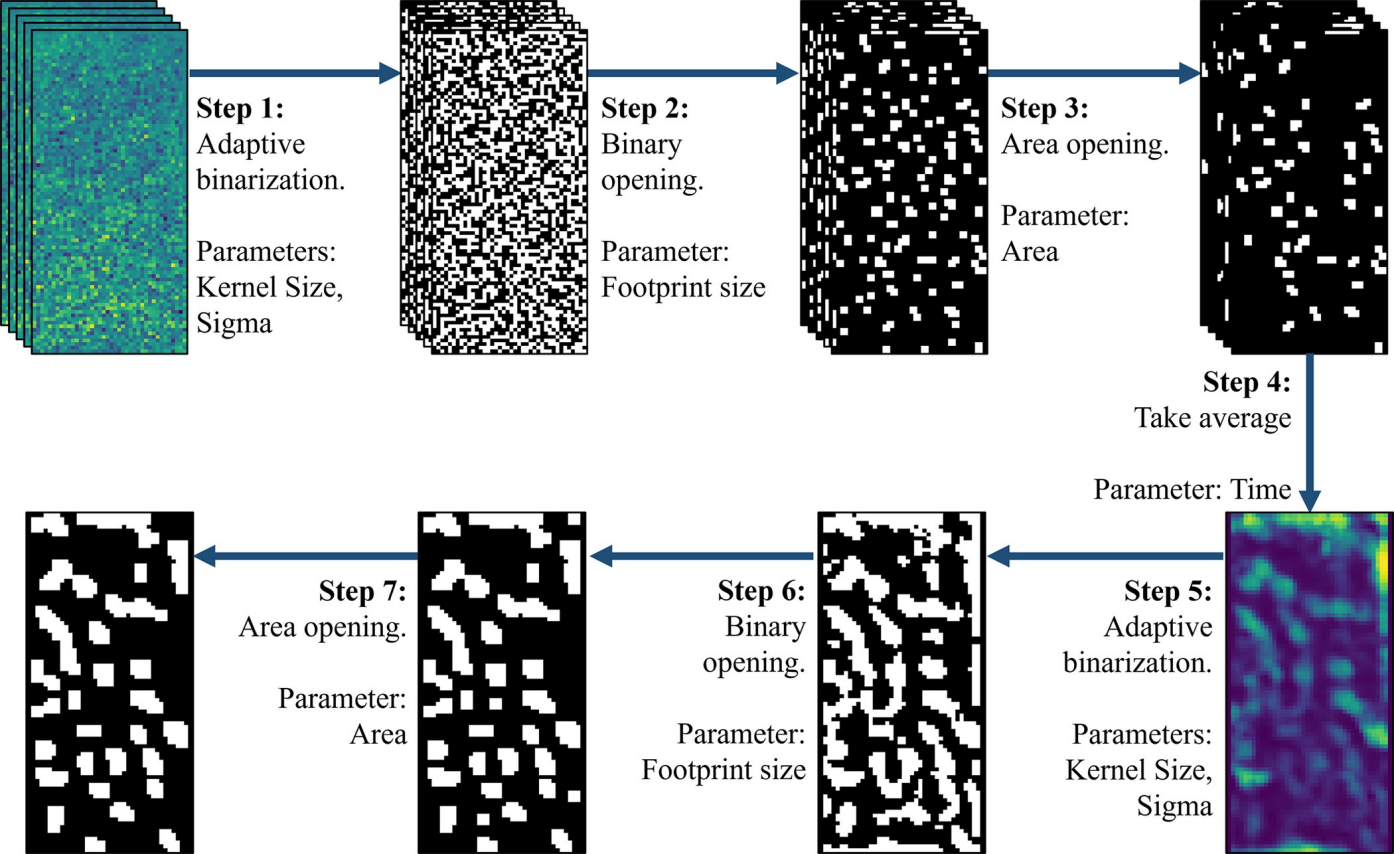
Overview of the ROI determination algorithm. A specified number of frames are adaptively binarized and then cleaned with a sequence of morphological opening and morphological area opening. The images are averaged, binarized again and cleaned with the same sequence of morphological processes.

We developed an implantable microimaging device for monitoring calcium imaging signals in the mouse brain. The imaging device is lensless and, consequently, the video (S1 Video) recorded by the device captured lacks definite features. Featureless ROI selection was conducted using adaptive binarization to find regions with high concentrations of fluorescence activity. The ROI selection algorithm (illustrated in Fig 2) consists of the following steps:

Step 1. Each frame is binarized adaptively with a Gaussian method. The operation can take different kernel sizes and sigma value for the Gaussian surface.
Step 2. Morphological opening is then applied to remove small features in the resulting images. Opening can take different footprint sizes.
Step 3. Morphological area opening is used to remove small features that were not removed with the previous opening. The tunable parameter is the size of the feature to be removed.
Step 4. The binarized and cleaned frames are averaged. The number of frames to be binarized can be changed.
Step 5. The average of the binarized frames is binarized again with a Gaussian method.
Step 6. The resulting frame is cleaned with morphological opening.
Step 7. Lastly, the remaining smaller features are removed by morphological area opening.

We modified each parameter and inspected the changes in the ROI mask to optimize the algorithm. There were 7 optimization parameters: sigma for the first and second adaptive binarization (σ_1_ and σ_2_), footprint size of the first and second opening (FP_1_ and FP_2_), area for the first and second area opening (A_1_, A_2_), and time for averaging.

In adaptive binarization with a gaussian method, a gaussian kernel centered at each pixel is convolved to that part of the image. The average of this matrix is then set as the threshold value for that pixel. Since the threshold value is different for each pixel, local maxima can be effectively identified. In addition, the sensitivity of the operation to local activity or noise can be tuned by the properties of the gaussian kernel, which is determined by the size and sigma parameter of the gaussian kernel. The first set of optimization parameters, σ_1_ and σ_2_, come from this operation. Increasing the sigma, or standard deviation, broadens the gaussian kernel which increases the contribution of the pixels within the kernel that are farther away from the center of the kernel.

Another parameter in adaptive binarization that was not included in the optimization is the size of the kernel. This increases the number of pixels that will be included in the threshold calculation, but initial results showed that this parameter did not affect ROI selection.

The next set of optimization parameters, FP_1_ and FP_2_, come from morphological opening. Morphological opening is the combination of two morphological processes, erosion followed by dilation. In the erosion step, a footprint is centered at each pixel and then the pixel value is set to 1 if all pixels in the footprint are equal to 1. In the dilation step, the footprint is centered at each pixel again and the pixel is set to 1 if any of the pixels in the footprint is equal to 1. The combination of these two steps typically removes features that are smaller than the footprint [22].

Subsequently, a set of optimization parameters comes from area opening which are A_1_ and A_2_. Area opening is a more straightforward operation which counts the area of each form in an image. The area parameter sets the threshold for determining if the form will be retained or removed from the image. Any shape with an area lower than the specified area is removed.

At the inception of the algorithm, adaptive binarization was applied to the average image of Ca^2+^ imaging videos and then cleaned by morphological image processing. Morphological opening was used to denoise the image and then morphological area opening was used to remove small (< 9 pixels) ROIs that could not be deleted by morphological opening. However, the Ca^2+^ traces from these ROIs were not significantly different from the Ca^2+^ trace of the whole image. Hence, adaptive binarization was applied to each image to prevent possible loss of information caused by averaging the video. In the current form of the algorithm, local maxima information is retained even if it may have been too low to be detected due to big changes in fluorescence in that area. Effectively, the local maxima in each frame are weighted equally and not lost due to averaging. Thus, the final optimization parameter is time or how many equivalent frames are necessary to accurately determine ROIs.

Changes in the ROI mask were quantified by calculating the average ROI area and then partial least squares regression was performed to model the how the parameters affect ROI size and, consequently, to enable size selection by setting specific algorithm parameters. In partial least squares regression, the predictor (X) and response (Y) variables are projected in hyperplanes that explain maximal variance. This leads to better predictive performance, particularly when there is multicollinearity between predictor variables, but complicates the interpretation of the regression model.

Both sigma parameters and the second area threshold correlated positively with ROI size while both footprint parameters correlated negatively. The first opening area did not have a strong correlation with the ROI area. This means that the isolated small features removed by this operation in each frame may be too few and is removed when the binarized frames are averaged or the second area opening (step 7) achieves the same effect. Time (number of frames used) also did not have a strong correlation with ROI area. This means that the features captured by the adaptive binarization closely resemble each other and, therefore, adding more frames do not significantly contribute to the ROIs. In addition, the parameters from the second binarization and opening typically had a bigger effect on ROI area than the first binarization and opening because the second operations are closer in sequence to the final ROI mask and more directly affect the selection process. Lastly, the intercepts of these models are not close to zero, which indicates some of the variation in ROI size is not explained by the variation in the algorithm parameters. Table 1 summarizes the coefficients calculated from the partial least squares regression.

**Table 1:** Coefficient of each parameter after assessing partial least squares regression to average ROI area.

Coefficient	Mouse 1	Mouse 2	Mouse 3
σ_1_	5.81	9.17	8.30
FP_1_	-6.65	-4.66	-4.17
A_1_	-0.91	0.01	0.07
σ_2_	11.96	17.63	14.29
FP_2_	-6.71	-8.66	-4.33
A_2_	3.27	4.47	4.09
Time	0.42	-0.30	2.91
Intercept	36.00	39.03	37.55

σ_1_, σ_2_ and A_2_ are positively correlated to average ROI area while FP_1_ and FP_2_ are negatively correlated. A_1_ and time are not strongly correlated with ROI area. The intercepts of the regression models are not close to zero and, therefore, parameter variations do not solely explain the changes in ROI size.

**Fig 3:**
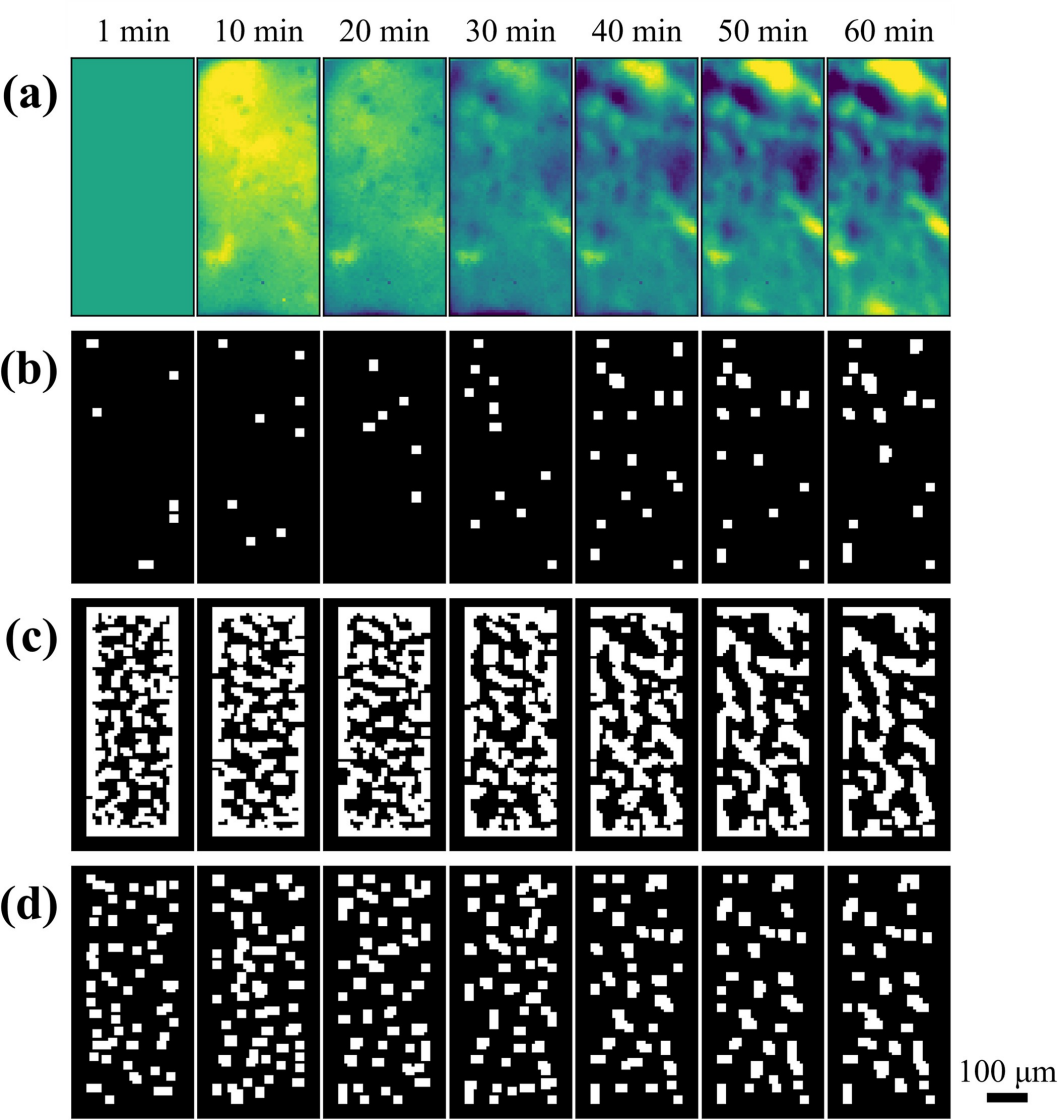
Comparison of ROI masks generated using different algorithm parameters. (a) Average image after 1, 10, 20, 30, 40, 50 and 60 minutes of Ca^2+^ imaging. (b-d) ROI mask generated after running the algorithm on the corresponding Ca^2+^ imaging data when the parameters are: (b) σ_1_ = 1.5, FP_1_ = 2, A_1_ = 0, **σ**_**2**_ **= 0.5, FP**_**2**_ **= 3**, A_2_ = 0, (c) **σ**_**1**_ **= 2.5,** FP_1_ = 2, A_1_ = 0, σ_2_ = 2.5, FP_2_ = 2, A_2_ = 0, and (d) **σ**_**1**_ **= 1.5, FP**_**1**_ **= 3**, A_1_ = 0, σ_2_ = 1.5, FP_2_ = 3, A_2_ = 0. The first set of images (a) illustrates a typical case of too few ROIs. The second set (b) exhibits a typical case of too big ROIs. The last set of images (c) exhibits uniformly distributed and sized ROIs.

The model had a low coefficient of determination (R^2^) of 0.6101 and relative prediction deviation of 1.602 so we opted to manually examine how the parameters affect the ROIs. Visual inspection of the ROI masks generated by the optimization reveals combinations of parameters that results in 1 of 3 types: sparse and small ROIs (Fig 3B), very large ROIs (Fig 3C), or small and uniform ROIs (Fig 3D). When σ_2_ = 0.5 and FP_2_ = 3, the features that pass through the binarization are smaller than opening footprint, so most features are deleted causing a sparse distribution of small ROIs (Fig 3B). In contrast, when σ_1_ = 2.5, most ROIs are overly large because the features that pass from each frame are large and further merge into larger features in the final binarization (Fig 3C and S1 Fig). Optimal ROI sizes were found when setting σ_1_ to 1.5 and FP_1_ to 3 where the ROIs are distributed uniformly throughout the imaging area and have consistent sizes (Fig 3D). A uniform distribution is important to prevent exclusion of active ROIs that have low intensity. Even though the number of ROIs is artificially increased, non-active ROIs can just be discarded at a later part in the pipeline.

The partial least squares optimization determined σ_1_ and FP_1_ to be less correlated to ROI size than σ_2_ and FP_2_. However, the optimal settings were found to be with a specific σ_1_ and FP_1_. This seems contradictory but this is due to the difference in optimization metrics. For partial least squares, the goal was to find the parameters that best predict average ROI size while uniformity was more important for the visual inspection. In addition, finer ROI size control was afforded by the weaker correlation of σ_1_ and FP_1_ so an optimal size was more easily found. The same size and uniformity may be found at a specific σ_2_ and FP_2_ that was not used during the optimization.

### 3.2. Validation with simulated data

We first evaluated the ROI algorithm with simulated Ca^2+^ imaging data. The datasets contained 30 randomly positioned neurons acting as point sources of light. In a 40 x 120-pixel field of view and taking the left topmost pixel as (0, 0), the neuron can be positioned between 5 to 35 in the x-axis and between 5 and 115 in the y-axis. The edges of the imaging area (5 pixels of columns and rows) are avoided because this is the noisiest part of device. Excitation light can enter through imperfections in the absorption filter or poor placement of the filter causing a noisy signal. The z-position or the distance from the imaging area can be between 4 to 8 pixels in terms of distance or equivalent 30 to 60 μm. The minimum distance for the neurons is 30 μm because based on previous experiments, most of the neurons from within 30 μm from the device are damaged and, therefore, cannot be imaged.

The videos contained 45,000 frames with 120 x 40 pixels per frame imitating typical behavioral experiments conducted with our devices. The first 9,000 frames (15 minutes) were designated as pre-stimulation frames, and the other 36,000 frames (60 minutes) were post-stimulation frames. During the pre-stimulation frames, all the 30 neurons have a 10% probability to spontaneously fire. In the post-stimulation frames, the 30 neurons are randomly assigned 3 patterns of activity. These patterns represent 3 simple and different firing patterns to demonstrate algorithm performance. Pattern 1 activity was high activity in the first 10 minutes, medium activity in the next 20 minutes and low activity in the last 30 minutes. In pattern 2 activity, high activity was shifted to 10 minutes after the stimulation. Unresponsive type of activity meant no change from spontaneous activity. High activity was set as 10 Hz firing rate with a 25% chance of bursting containing 2 to 5 spikes chosen randomly. Medium activity involved 10 Hz firing rate with a 1 in 15 (6.66%) chance of bursting. Lastly, Low activity was 5 Hz firing with a 1 in 20 chance (5%) chance of bursting.

**Fig 4:**
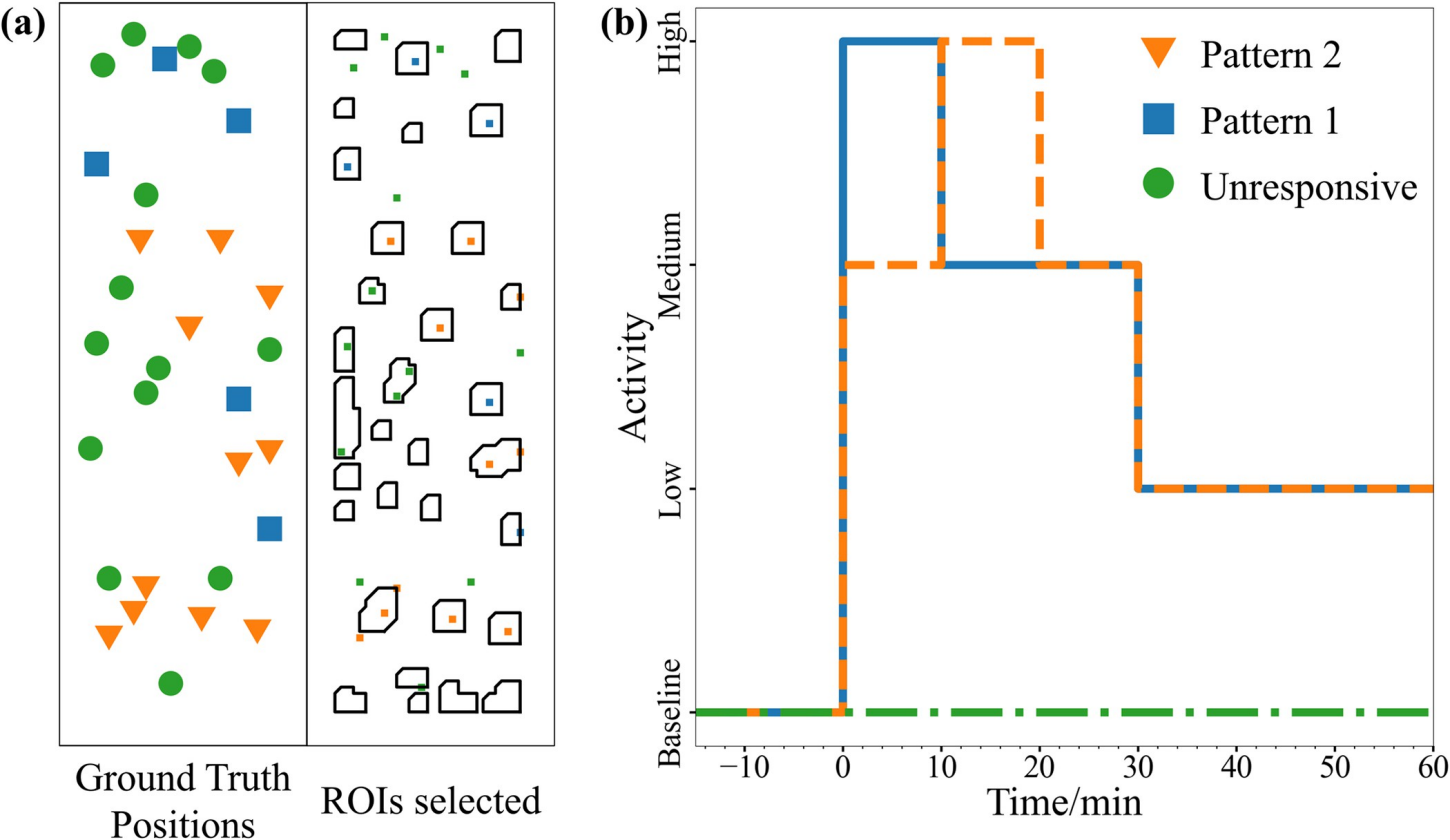
Comparison of ground truth position of simulated neurons with different firing patterns and ROI selection by algorithm. (a) The position of each simulated neuron in the simulated field of view is displayed with its corresponding activity pattern. (b) Pattern 1 neurons exhibit high activity in the first 10 minutes, medium activity in the next 20 minutes, and low activity in the last 60 minutes. In pattern 2 neurons, the high activity is shifted to the second 10 minutes and the first 10 minutes is medium activity. Unresponsive neurons maintain baseline activity throughout the simulation.

We evaluated whether the algorithm can correctly identify ROIs in the simulated dataset (raw video in S2 Video). Fig 4 shows the selected ROIs and the ground truth positions of simulated neurons in an example simulation. It can be observed that the algorithm did spuriously increase the number of ROIs because there are ROIs without simulated neurons inside the region. Some unresponsive neurons are even captured by ROIs. Nevertheless, the number of ROIs can be decreased by grouping them into active and non-active ROIs by principal components analysis (PCA) and k-means clustering, as stated previously.

Before clustering, the signal quality was enhanced with spectral subtraction by treating non-ROI pixels as noise. Short-time Fourier transform (STFT) was conducted on each ROI trace and then the noise STFT spectrogram was subtracted from each ROIs’ spectrogram and an inverse-STFT was performed. STFT can be used to describe a time series into its spectral components particularly when the time series is non-stationary. Fluorescence activity is expected to significantly change with time and so will the noise component of the signal due to the multiple stray light sources. Hence, the signal is non-stationary and was improved with STFT spectral subtraction.

**Fig 5:**
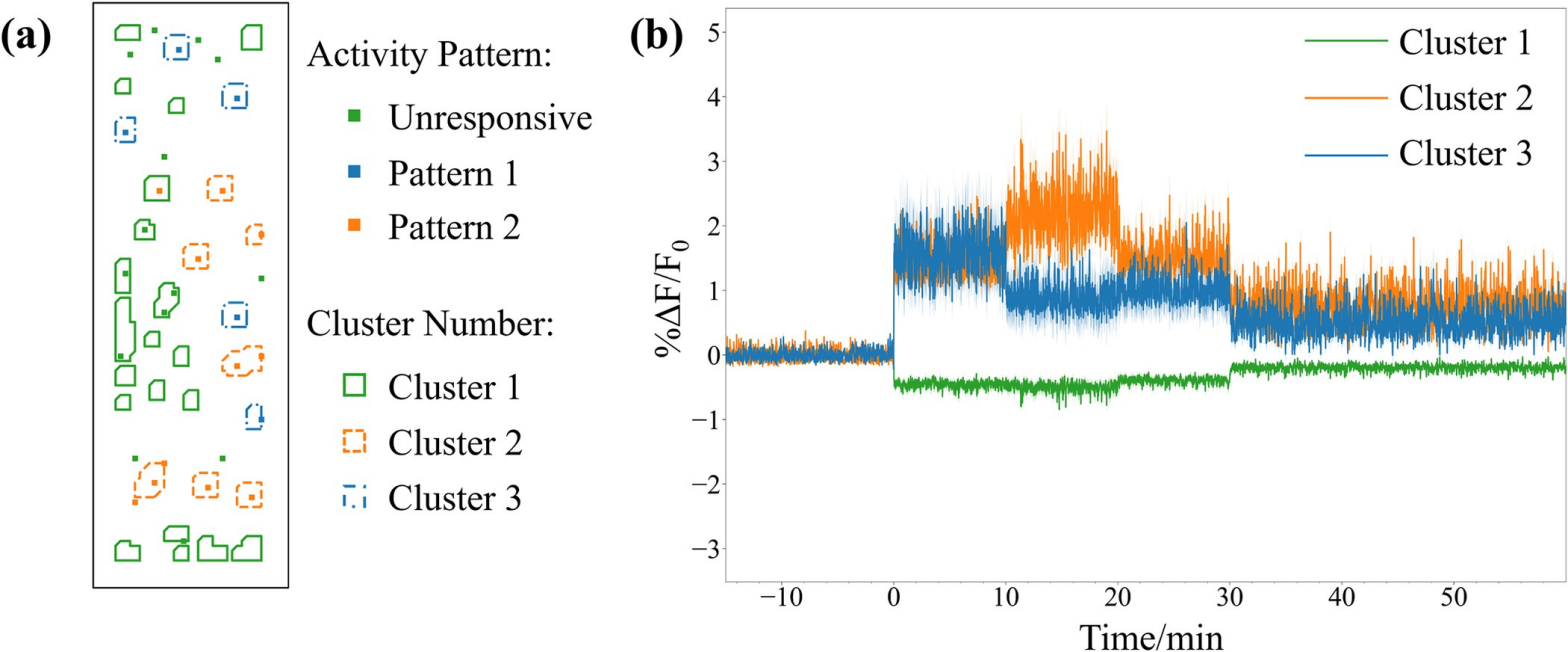
Clustering of ROIs and average trace of each ROI cluster. (a) Ground truth position of simulated neurons with ROIs selected by the algorithm and clustered with PCA and k-means. (b) Average ΔF/F_0_ of clusters 1 to 3 which closely resembles the three ground truth activity patterns.

We then classified the spectrally subtracted traces into active and non-active ROIs with PCA and k-means. Principal components were restricted to number of components that account for 95% of the variance in the data. We performed k-means clustering and used the average silhouette score to determine optimal number of clusters [33]. In the example simulation, the optimal number of clusters was 3 as shown in Fig 5A. The black-colored clusters contained ROIs with no neurons in them or unresponsive neurons. The blue cluster of ROIs contained neurons with pattern 1 activity while the orange cluster contained neurons with pattern 2 activity. Thus, the ROIs can be classified into its activity pattern using PCA with k-means. Furthermore, the average fluorescence activity of the clusters closely resembles the fluorescence activity of the simulated neurons (Fig 5B).

**Fig 6:**
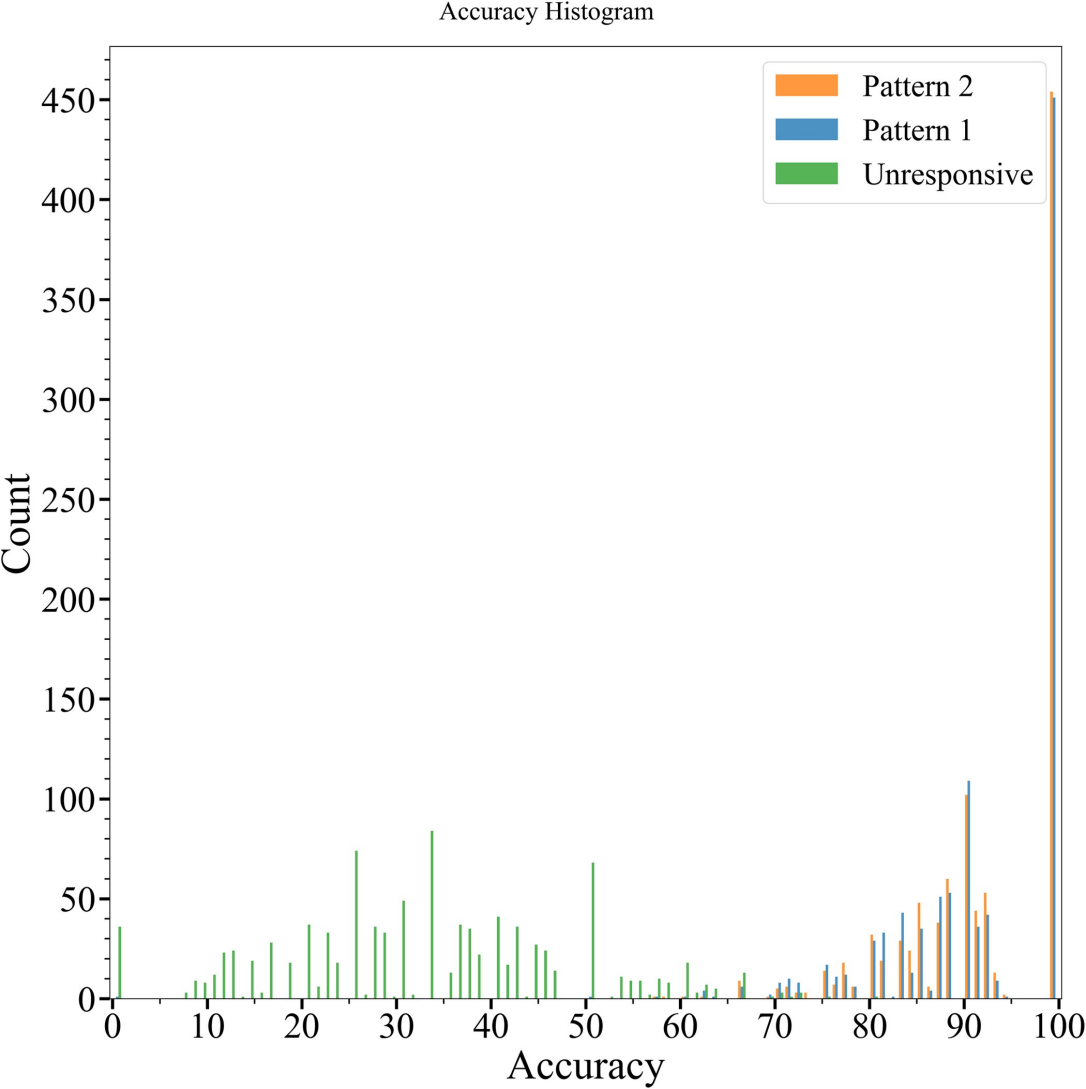
Accuracy of ROI algorithm in detecting simulated neuron position over 1000 simulations. For each simulation, the number of neurons tagged as ROIs was counted and divided by the total number of neurons of that activity pattern. This histogram shows that the neurons that are not detected are the inactive neurons. Over 400 simulations correctly marked 100% of the active neurons.

We then evaluated the accuracy of the algorithm across 1000 simulations. The number of neurons of each activity pattern that was enclosed in an ROI was counted and divided by the total number of neurons of that activity pattern. Over 400 of these simulations captured 100% of the active neurons as shown in the histogram in Fig 6. In a simulation by de Kraker et al., 238, 171, and 160 neurons out of 514 ground truth neurons were found with CNMF, Suite2p, SpecSeg, respectively. This is equivalent to 46, 33 and 31% of the ground truth neurons being detected. In addition, 90% of the active neurons are captured in over 700 simulations (S2 Fig). The most likely cause for this decrease in accuracy is the distance of the neuron from the sensor. As shown in S3 Fig, the number of undetected active neurons increases with increasing distance from the sensor.

### 3.3. Validation with freely moving experimental data

**Fig 7:**
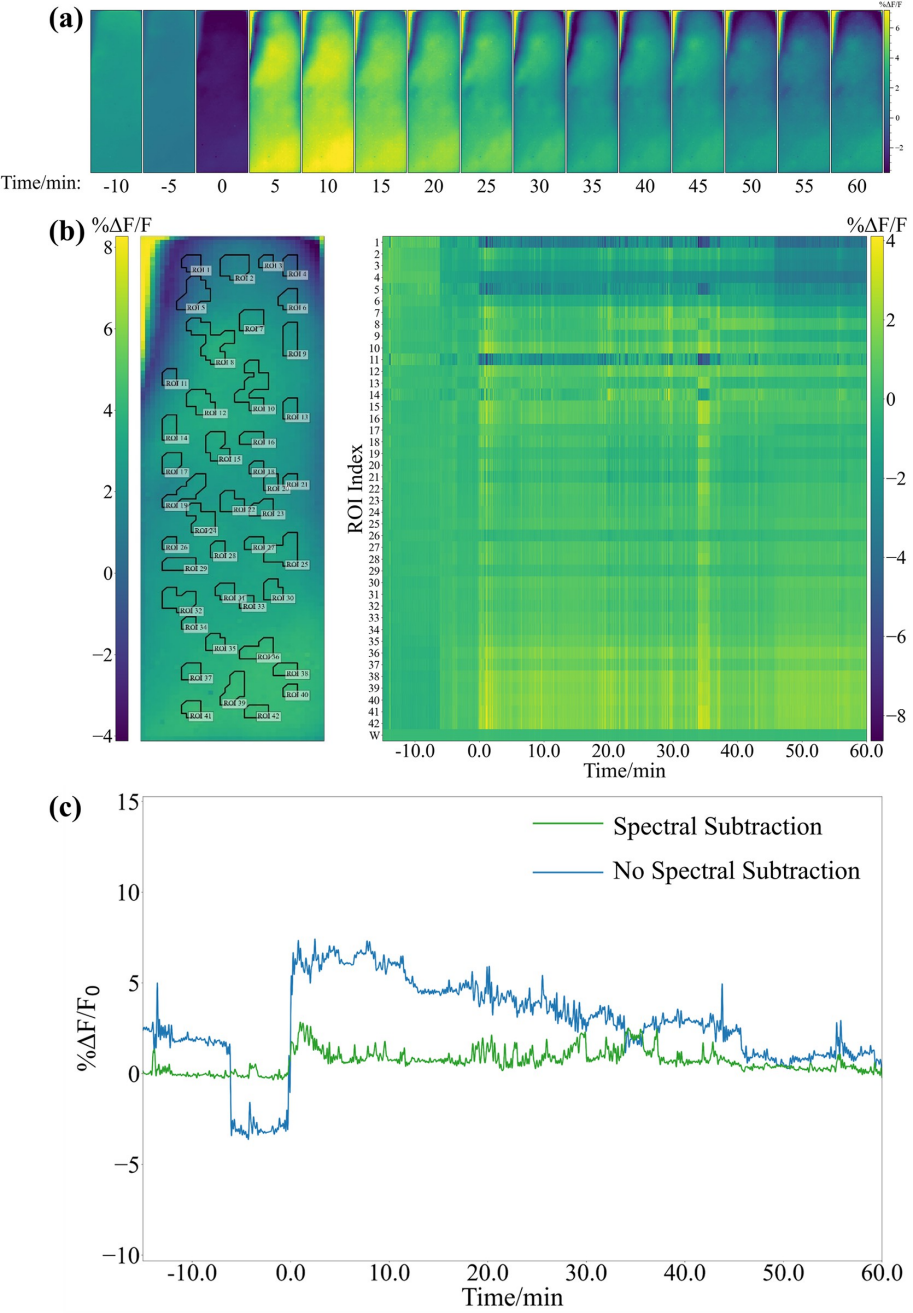
DRN activity recorded with CMOS-based device after formalin injection. (a) Average DRN fluorescence frame every 5 minutes shows clear difference between prestimulation and poststimulation frame average. (b) Average fluorescence time series of each ROI shows that difference in activity among ROIs. (c) Difference between %ΔF/F_0_ traces with and without spectral subtraction.

**Fig 8:**
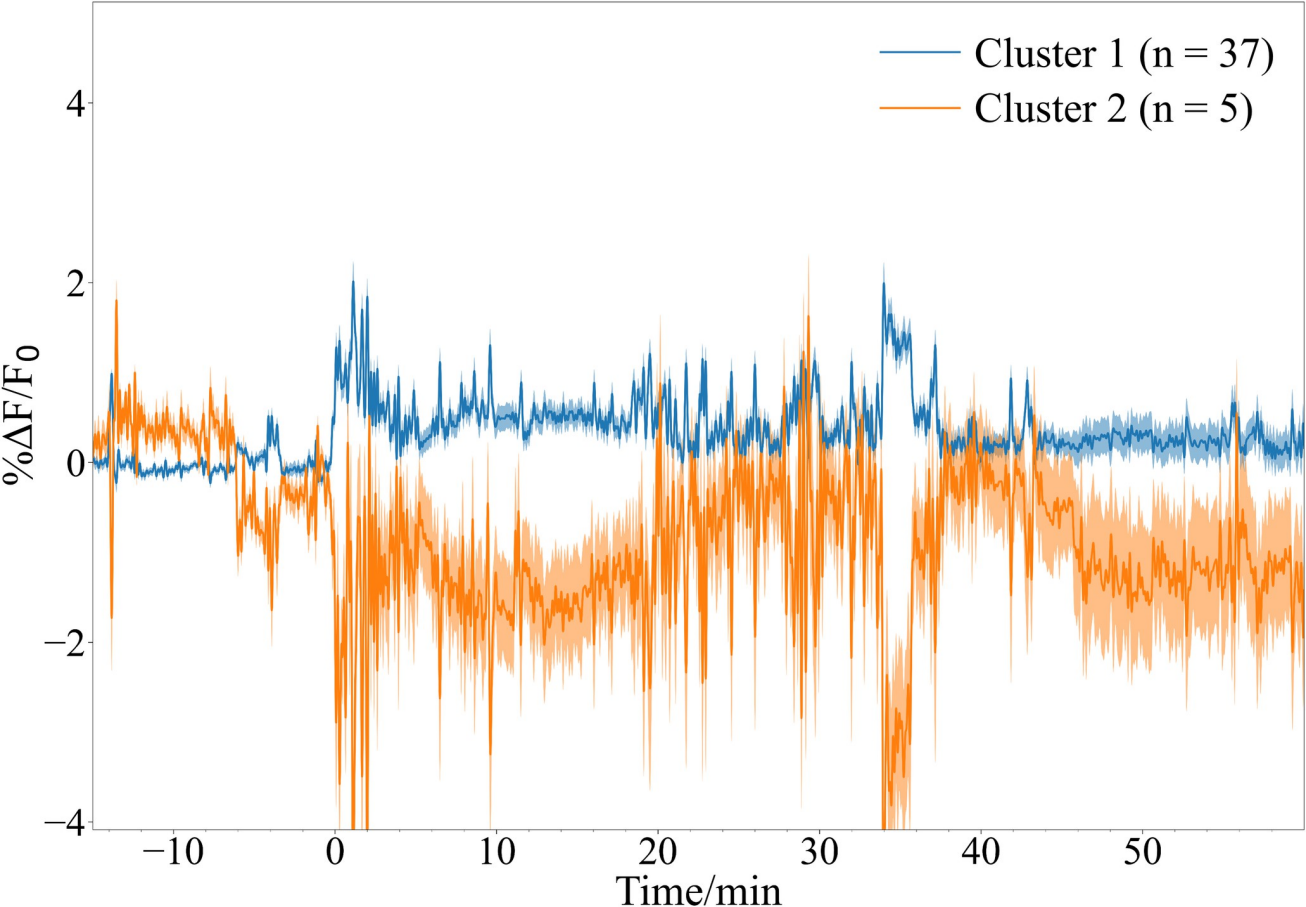
Average trace of each ROI cluster at maximum silhouette score. The ROIs were clustered with PCA and k-means to functionally differentiate the ROIs between active and non-active. Clear differences between the activity patterns of each cluster can be observed proving the effectiveness of the grouping.

We then evaluated the ROI algorithm with Ca^2+^ recordings obtained from our device in a freely moving mouse experiment. Left DRN activity was recorded after injection of paraformaldehyde into a mouse’s right dorsal hind paw surface to demonstrate observable changes in fluorescence activity. Fig 7A shows the average image every five minutes during the experiment. Fig 7B illustrates the Ca^2+^ traces represented in pseudocolor from each ROI determined by the algorithm. The signal improvement caused by spectral subtraction is demonstrated in Fig 7C. Non-stationary noise is present in all pixels due to the lensless nature of the devices. Since the ROI pixels contain the relevant fluorescence data, noise was reduced by subtracting the spectral information of non-ROI pixels which contain mostly noise. After spectral subtraction, fluorescence traces were clustered with PCA and k-means. As shown in Fig 8, the ROIs can be effectively separated into active and non-active ROIs.

**Fig 9:**
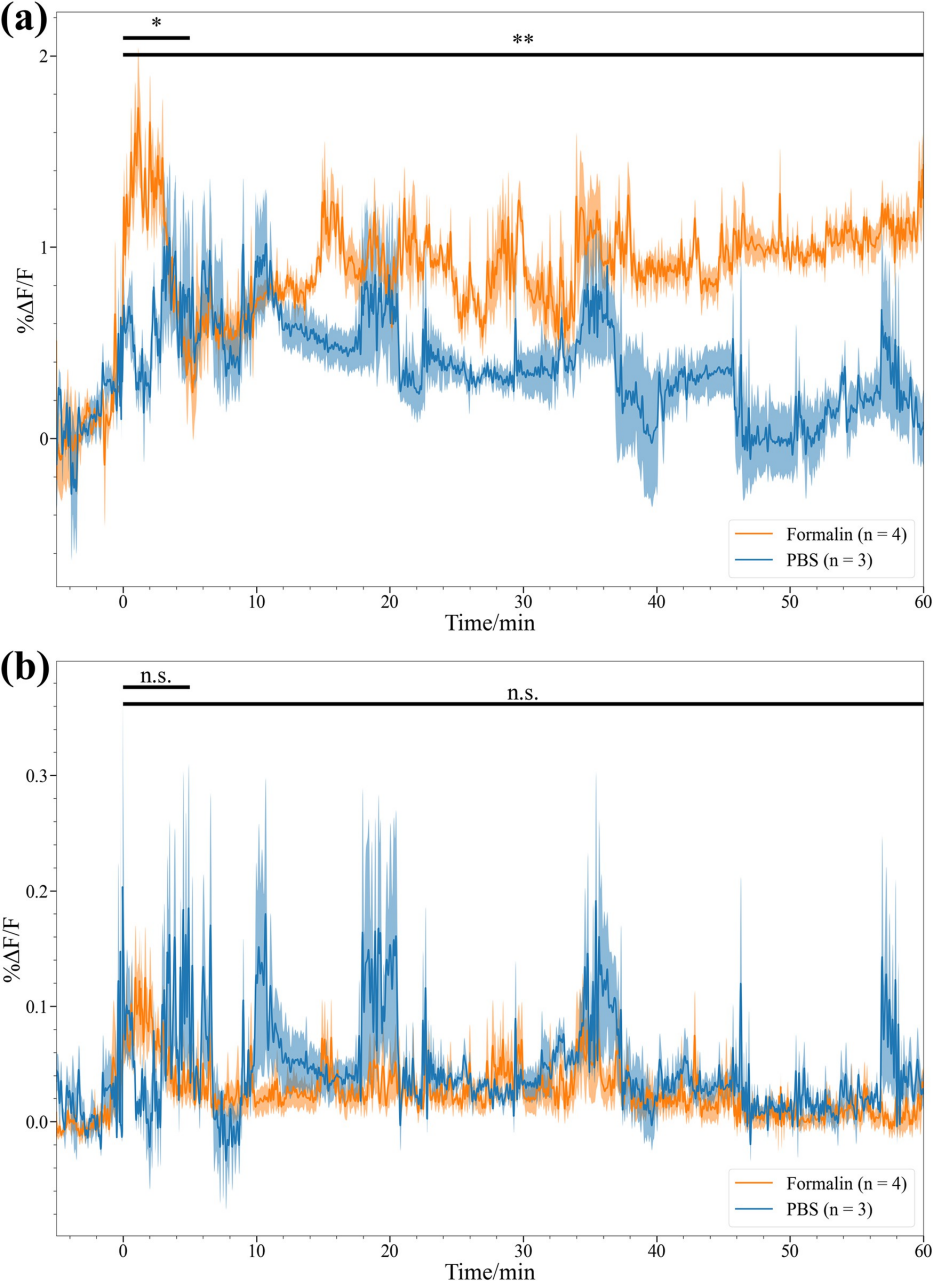
Groupwise comparison of formalin- and PBS-injected mice’s DRN activity using ROI algorithm and whole frame averages. Average trace of highest activity ROI clusters (a) of each mouse in each treatment group compared to the average of the whole image (b). The average intensity of the formalin group is higher than PBS group 5 minutes after the stimulation and even throughout the 60-minute experiment. This is not the case when analyzing the data using only whole image averages.

For control experiments, mice were injected with phosphate buffered saline (PBS) to not trigger activity in the DRN. We then compared the average fluorescence activity of the left DRN after injection of formalin or PBS into the right dorsal hind paw surface of mice. Statistically higher DRN activity was observed for mice injected with formalin than PBS in the first 5 minutes (p < 0.05) and whole 60 minutes (p < 0.01) after stimulation (Fig 9A). The cluster with the highest average fluorescence for each mouse was designated as active and used for these comparisons. Only one cluster was used, and the traces were averaged to avoid biasing the group to the mice with more ROIs or more active clusters. In addition, these differences are not observed when using the average fluorescence traces of the whole frame (Fig 9B) proving the utility of the ROI selection algorithm. Furthermore, the %ΔF/F_0_ values of the ROI traces are also an order of magnitude higher than whole frame traces.

## 4. Discussion

Measuring neuron action potential with electrophysiological technique is the gold standard for observing brain activity. However, calcium imaging offers unique advantages such as high spatial resolution, simultaneous recording of multiple locations and neuron-type specificity. In our laboratory, we developed an ultrasmall implantable microimaging device for calcium imaging to decrease implantation damage and promote natural mouse behavior. Additionally, multi-probe implantations have been conducted to simultaneous observe multiple regions in the mouse brain [17, 19, 22]. Integration with other modes of sensing such as fast-scan cyclic voltammetry [34] and electrophysiology [35, 36] have also been pursued to capture a more comprehensive recording of brain activity. In this work, we improve on the imaging devices by designing an automatic ROI selection algorithm.

### 4.1. Parameters optimization

S1 Video shows what a typical video taken with the microimaging devices looks like. There are clear areas in the video where the fluorescence intensity increases or decreases which can represent changes in neural activity. However, the edges of these fluorescent ‘forms’ are ambiguous and, consequently, it is difficult to define regions of interest (ROI) in the video. The ambiguity of the boundaries arises from the lensless nature of the device. Light rays coming from GECI molecules are not focused into the imaging area and, thus, the signal from each neuron cannot be localized and just mixes with each other. A fluorescence gradient also exists in all the frames of the video due to the uneven LED excitation. Hence, adaptive binarization was utilized to identify local maxima in these frames.

Adaptive binarization calculates the threshold value for each pixel in an image based on the brightness value of the surrounding pixel. This lessens the effect of the brightness gradient from the LED’s uneven illumination. Therefore, local fluorescence maxima from increased Ca^2+^ can be located. Initially, adaptive binarization was applied to the only average image of whole imaging experiments and then cleaned by morphological image processing. The processes used were morphological opening, which smoothens the contour of features in an image, and morphological area opening, which removes features smaller than a certain area. However, the Ca^2+^ traces from these ROIs were not significantly different from the Ca^2+^ trace of the whole image. Hence, frame-by-frame adaptive binarization was conducted to decrease information loss caused by averaging the video.

The next step was to optimize the algorithm as each operation had a parameter that needed to be defined. Although the effect of each parameter could be easily understood when applied to a single image, it was unknown how it will affect the ROI mask that will be generated by the algorithm.

In adaptive binarization, the parameters are kernel size and sigma value. Both parameters affect the shape of the gaussian kernel that will be convolved with the image. A larger kernel size increases the number of neighboring pixels that will be considered for setting the threshold. This causes the binarization to be less sensitive to local noise since the effect will be averaged over more pixels. Similarly, decreasing the sigma value concentrates the gaussian kernel to the center which decreases the weight placed on neighboring pixels. Thus, decreasing the sigma decreases sensitivity to noise in the image. However, this may cause finer details in the image to be missed.

In morphological opening, the parameter was footprint size. Footprint refers to the kernel that scans through the image to determine if the pixel will be 0 or 1. Features that are smaller than the footprint size are typically removed in the image [37]. Area opening simply removes features that are smaller than a specified area. This was initially used to remove very small features that remained after opening but it was unclear whether it was important in the algorithm.

PLSR showed which parameters caused an increase or decrease in the average ROI size. However, the model cannot be effectively used to control the size of the ROI due to low coefficient of determination of 0.6101 and relative prediction deviation of 1.602. Thus, optimization was judged by ROI patterns observed with specific combinations of algorithm parameters. Setting σ_1_ to 1.5 and FP_1_ to 3 resulted in uniformly distributed ROIs with consistent sizes. This is important to uniformly present local maxima throughout the whole imaging area which prevents exclusion of active ROIs that have low intensity. The optimized algorithm takes only approximately 12 seconds to analyze 60 minutes’ worth of frames (36,000 frames).

### 4.2. Validation with simulated and experimental data

Validation with simulated data revealed that there are numerous ROIs that contain no neurons inside. This similar problem was observed with other ROI selection methods. In the report of de Kraker *et al*., 1079, 573 and 352 ROIs were detected by CNMF, Suite2p and SpecSeg, respectively, in a simulated data containing 514 ground truth neurons [28]. To remedy this, the ROIs were clustered together by activity to differentiate active ROIs from non-active ROIs. K-means clustering was performed following feature extraction with PCA. PCA simplifies a dataset into a representation that captures the maximum variance of the dataset. We performed PCA to avoid biasing the clustering metric to only the first few minutes of the data where activity differences are obvious. This prevents exclusion of low intensity activities. We then performed k-means clustering to group the principal components of the ROI time series. K-means clustering is an iterative algorithm that groups similar data points based on Euclidean distance. After designating the number of k-clusters, a random centroid for each cluster is initiated. The nearest centroid to each data point is designated as its cluster and the distance from the centroid is calculated. The centroid positions are then adjusted to minimize the average distance and the distance is measured again. The iteration is stopped once the centroid no longer shifts significantly. Using PCA and k-means clustering, the ROIs were partitioned effectively into the different activity patterns. The average fluorescence activity of the ROIs in a cluster showed great correlation with ground truth activity patterns.

Validation with experimental data showed statistically significant differences between control and stimulation groups in the first 5 minutes (p < 0.05) and the whole 60 minutes (p < 0.01) after stimulation. These differences are not observed when using the average fluorescence traces of the whole frame proving the utility of the ROI selection algorithm. Furthermore, comparisons were done using only the clusters with the highest average fluorescence for each mouse to avoid skewing the average to the mouse with more ROIs. However, it is worth noting that distinct activity patterns can be observed when the time series are clustered into 6 clusters which is a local maximum in the Silhouette score plot (S4 Fig). These clusters of ROIs could indicate different populations of neurons captured in the imaging area. Hence, in the future, the ROI algorithm can be used for experiments exploring the dynamics between different neural populations.

## 5. Conclusion

In this study, we report the development of an ROI selection algorithm for lensless microimaging devices. The changes to the average ROI area when changing algorithm parameters were assessed with partial least squares regression. The sigma values typically increased the average size of the ROIs while the footprint size of the morphological opening typically decreased the average size of the ROIs. We found uniformly distributed and sized ROIs when setting the first sigma value to 1.5 and the first footprint size to 3. We validated the algorithm with simulated datasets and found high accuracy detection of simulated neurons. We then demonstrated that using the ROI algorithm, statistically significant differences were found between the DRN fluorescence activity of mice injected with formalin and PBS.

The ROI algorithm has shown promising results in implantable lensless Ca^2+^ imaging devices. We believe that our discovery of the effects of the parameters in the ROI mask could pave the way for its application in lens-based Ca^2+^ microimaging systems which can also from uneven illumination. The principles behind the method can also be generalized beyond calcium microimaging to other fields requiring precise ROI detection and analysis.

## Supporting information

S1 Fig ROI masks generated when using 2.5 σ1. Compared to using optimal parameters which result in uniformly sized and distributed ROIs (a), using 2.5 σ_1_ results in ROIs that are too large (b-e). This is particularly evident when only 10–30 minutes of data is used. The parameters for each of the masks below are as follows: σ_1_ = 1.5, FP_1_ = 3, A_1_ = 0, σ_2_ = 1.5, FP_2_ = 3, A_2_ = 0, σ_1_ = 2.5, FP_1_ = 2, A_1_ = 0, σ_2_ = 2.5, FP_2_ = 2, A_2_ = 0, σ_1_ = 2.5, FP_1_ = 3, A_1_ = 0, σ_2_ = 2.5, FP_2_ = 3, A_2_ = 0, σ_1_ = 2.5, FP_1_ = 3, A_1_ = 0, σ_2_ = 2.5, FP_2_ = 3, A_2_ = 0, σ_1_ = 2.5, FP_1_ = 2, A_1_ = 0, σ_2_ = 1.5, FP_2_ = 2, A_2_ = 0.
(TIF)

S2 Fig Probability density of each bin of accuracy. (TIF)

S3 Fig Distance of each undetected neuron from the imaging sensor in 1000 simulations. The number of undetected neurons increases with distance from the sensor.
(TIF)

S4 Fig Average trace of each ROI cluster when clustering into 6 clusters. (TIF)

S1 Video Example of in vivo calcium imaging recording taken with implantable CMOS-based imaging device. (MP4)

S2 Video Example of simulated calcium imaging dataset. (MP4)
