# Supplementary figures and images for: Region of interest determination algorithm of lensless calcium imaging datasets
Source: PLoS One. 2024 Sep 17;19(9):e0308573. doi: 10.1371/journal.pone.0308573 (PMC11407621; doi:10.1371/journal.pone.0308573)

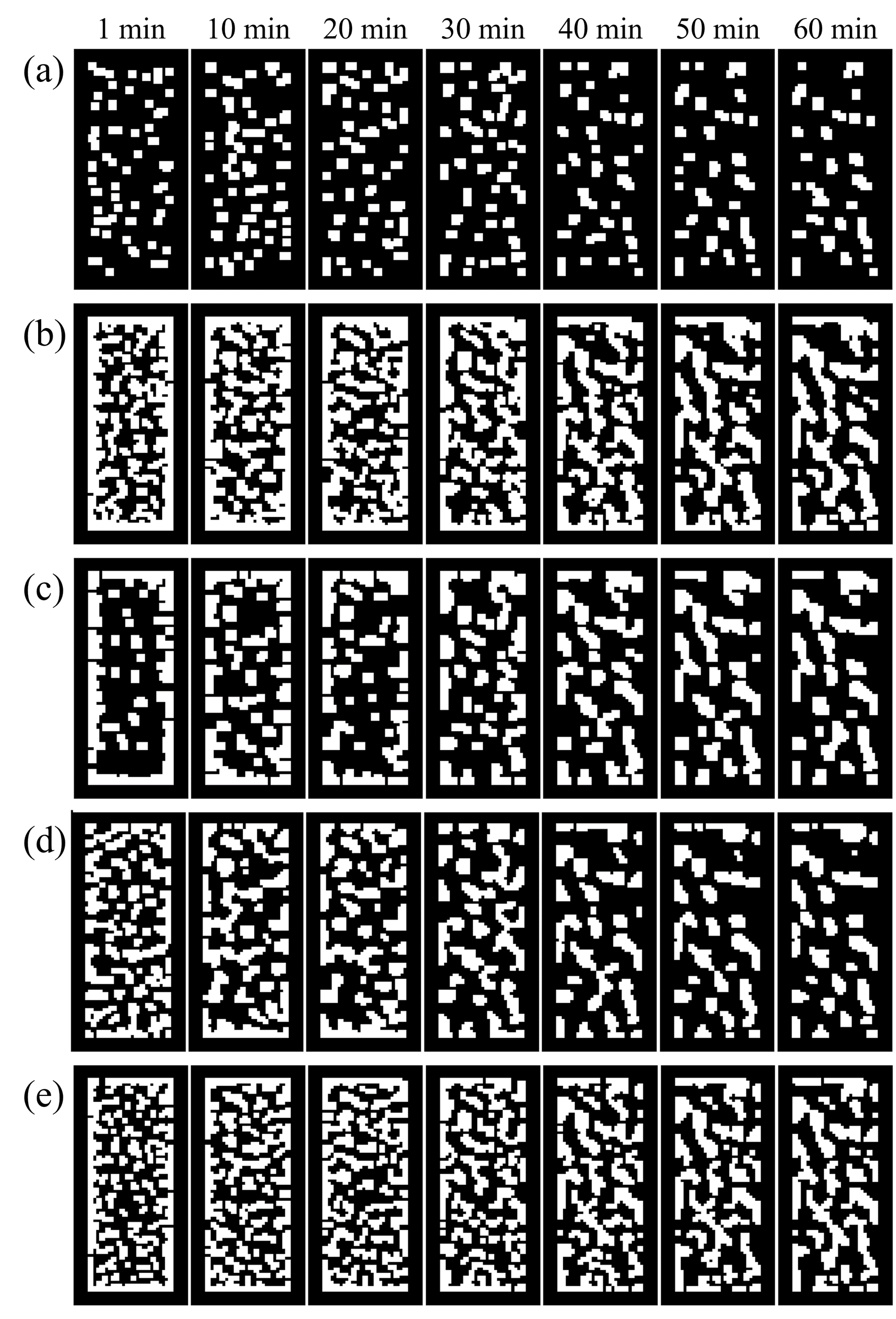

Supplement: S1 Fig — Compared to using optimal parameters which result in uniformly sized and distributed ROIs (a), using 2.5 σ1 results in ROIs that are too large (b-e). This is particularly evident when only 10–30 minutes of data is used. The parameters for each of the masks below are as follows: σ1 = 1.5, FP1 = 3, A1 = 0, σ2 = 1.5, FP2 = 3, A2 = 0, σ1 = 2.5, FP1 = 2, A1 = 0, σ2 = 2.5, FP2 = 2, A2 = 0, σ1 = 2.5, FP1 = 3, A1 = 0, σ2 = 2.5, FP2 = 3, A2 = 0, σ1 = 2.5, FP1 = 3, A1 = 0, σ2 = 2.5, FP2 = 3, A2 = 0, σ1 = 2.5, FP1 = 2, A1 = 0, σ2 = 1.5, FP2 = 2, A2 = 0. (TIF) [file pone.0308573.s001.tif]

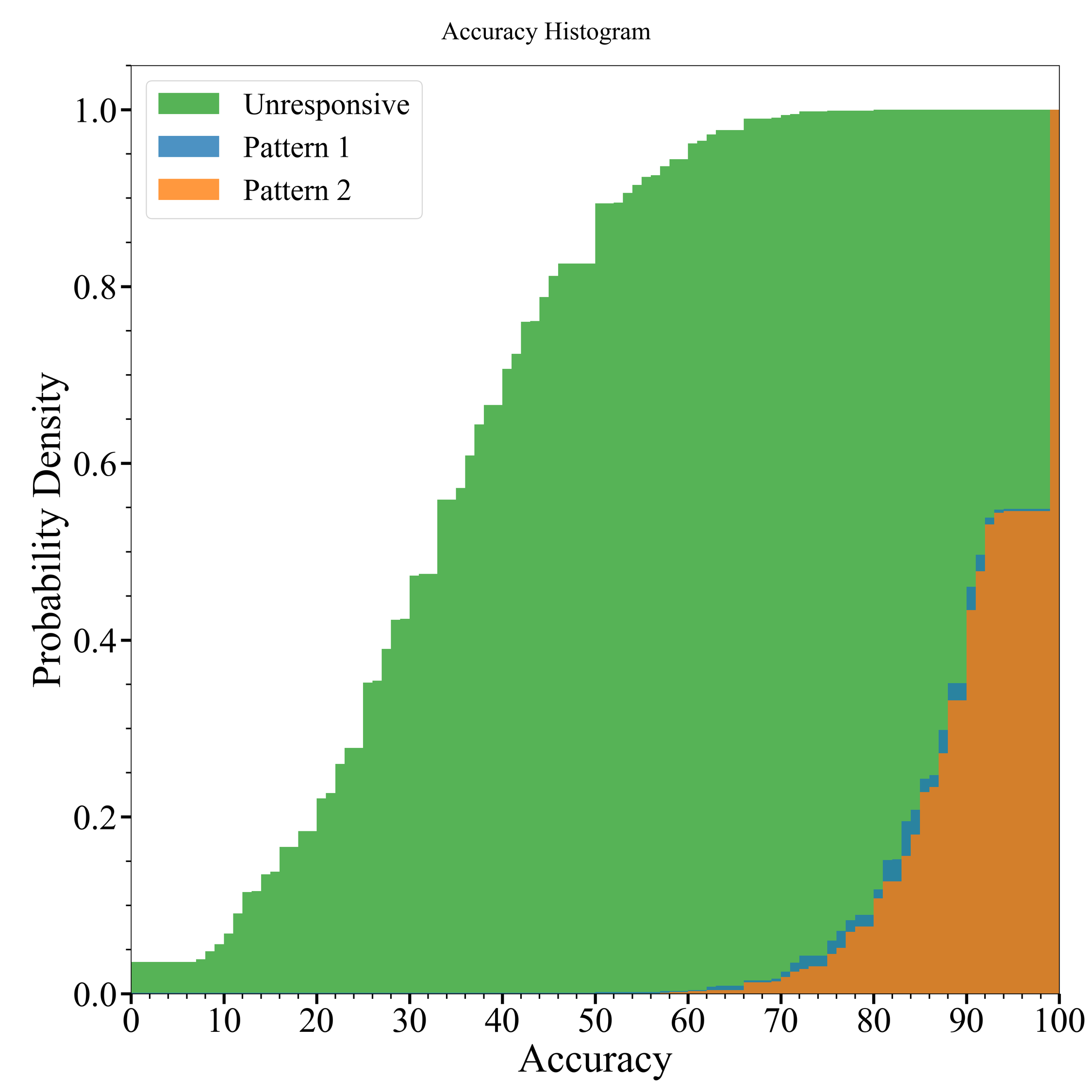

Supplement: S2 Fig — (TIF) [file pone.0308573.s002.tif]

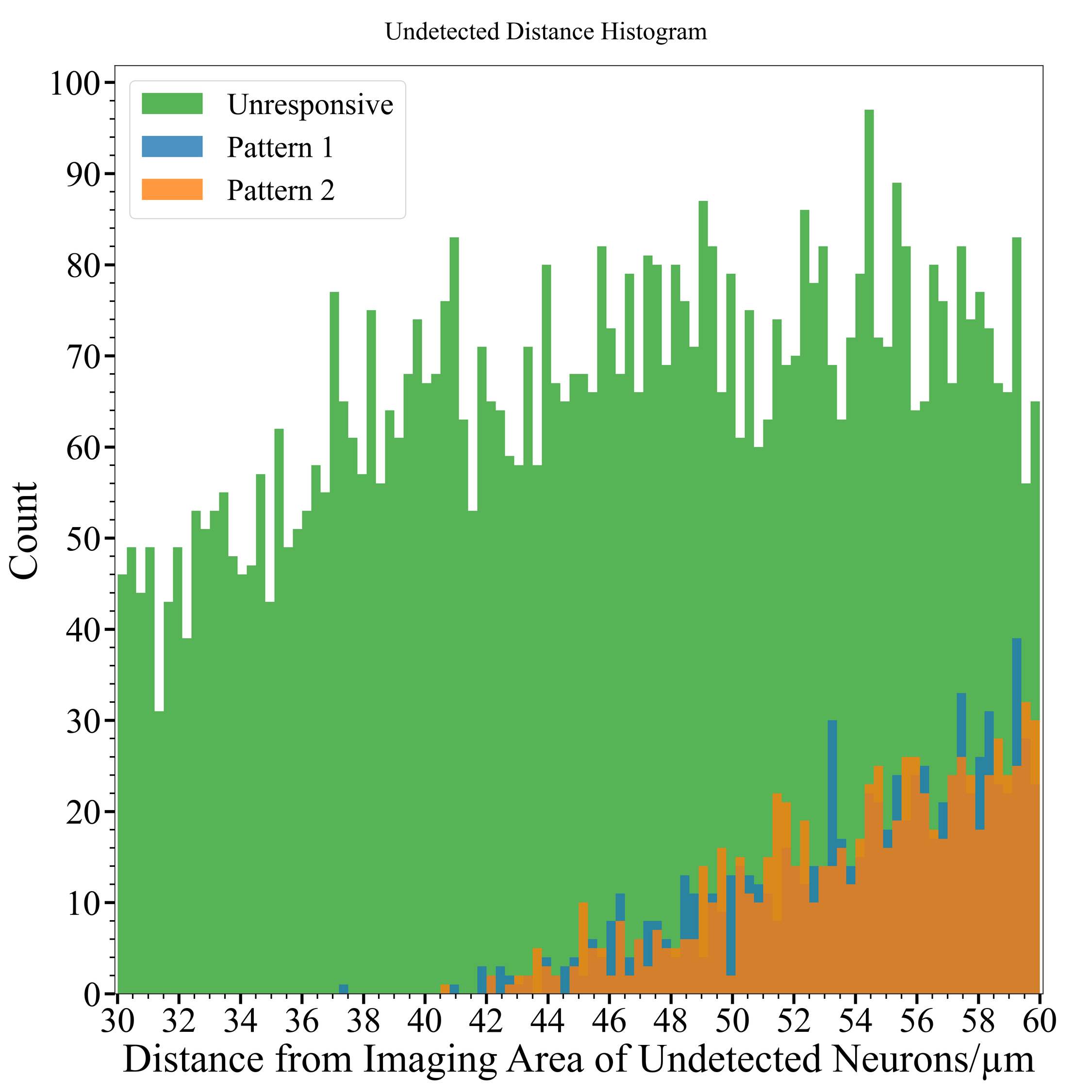

Supplement: S3 Fig — The number of undetected neurons increases with distance from the sensor. (TIF) [file pone.0308573.s003.tif]

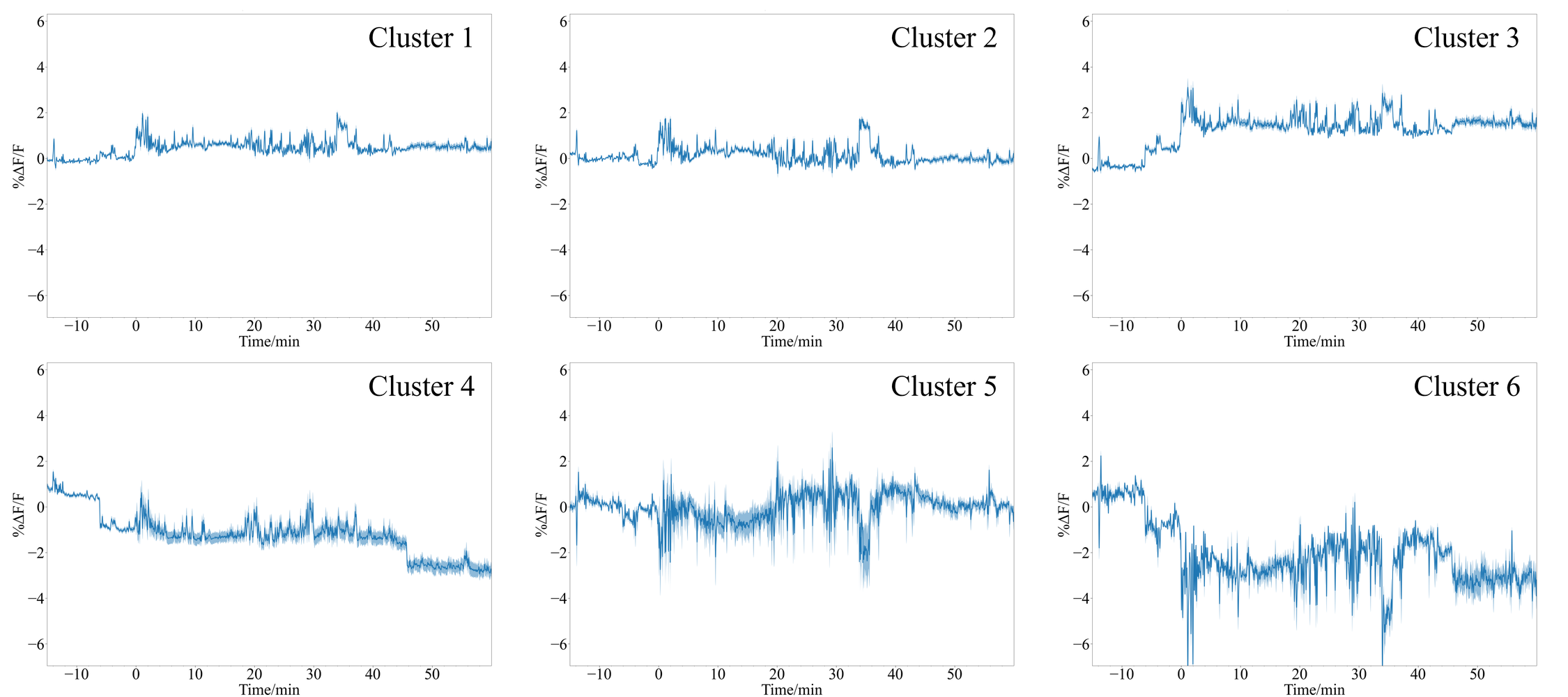

Supplement: S4 Fig — (TIF) [file pone.0308573.s004.tif]
